# MR and PET/CT imaging in diagnosis of large pelvic masses

**DOI:** 10.1186/1470-7330-15-S1-P48

**Published:** 2015-10-02

**Authors:** S De Luca, MF Grana, E Casalini Vañek, L Tolkachier, L Alarcon, E Eyheremendy

**Affiliations:** 1 Hospital Aleman, Buenos Aires, Argentina

## Learning objectives

Discuss possible causes of pelvic masses in female patients.

Discuss utility and clinical applications of MRI combined with PET/CT in the management of these patients.

Illustrate cases presented as large pelvic masses, of gynaecological and non gynaecological origin.

## Content organisation

MRI and PET/CT techniques.

Causes of pelvic masses in female patients.

Specific MRI and PET/CT findings.

Sample cases and mimics.

## Conclusion

The combined or isolated use of MR and PET / CT demonstrated to be useful in detecting gynaecological pathologies and in most cases it also allowed the identification of the organ of origin, extent and staging of the lesion.

PET / CT is a useful tool in cases where the assessment of MR is not enough.

